# Supplementary material for: Lost in translation: no effect of repeated optogenetic cortico-striatal stimulation on compulsivity in rats
Source: Transl Psychiatry. 2021 May 24;11:315. doi: 10.1038/s41398-021-01448-x (PMC8144623; doi:10.1038/s41398-021-01448-x)
Supplement: Supplementary file 1 — Supplemental material [file 41398_2021_1448_MOESM1_ESM.pdf]

Supplementary Material

Supplementary Results

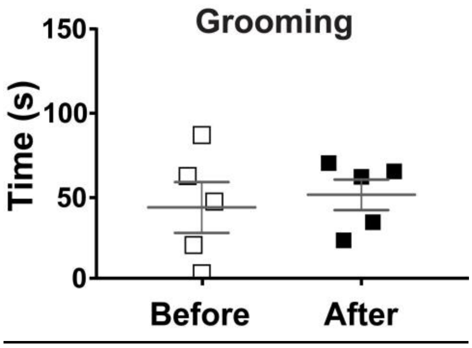

Supplementary Fig. 1. OFC-VMS repeated opto-stimulation did not increase self-grooming behavior in a second cohort of rats. Total duration of self-grooming behavior assessed for 5 min before and after the OFC-VMS opto-stimulation protocol. There was no change in grooming expression ($p = 0.76$). Markers represent individual animals; bars indicate mean and SEM. n = 5.

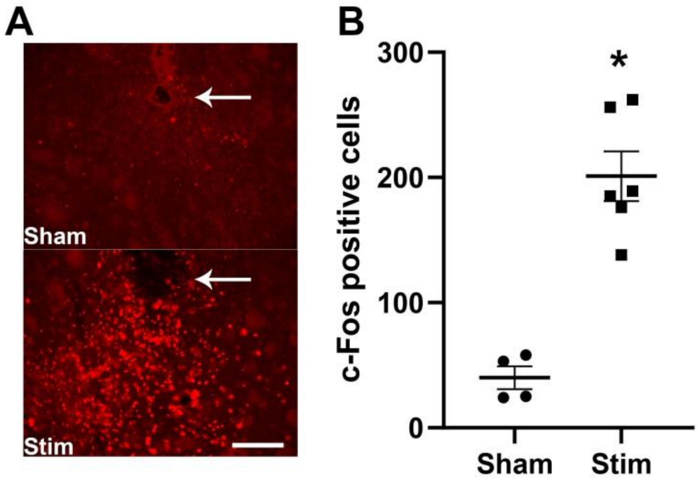

Supplementary Fig. 2. Electrical stimulation increased c-Fos expression in the VMS. A) Representative images of slices of the VMS demonstrating electrical-induced neural activation. c-Fos immunostaining (red). Localization of the tip of the electrodes (arrows). Scale bar 200 µm. Electrical stimulation was applied at 130 Hz for 60 min. **B)** Quantification of c-Fos-positive cells in the VMS of electrical-stimulated animals compared to sham-stimulated controls. Markers represent individual animals; bars indicate mean and SEM. There was an increased c-Fos expression in the VMS after electrical stimulation compared to controls ($\exp(\beta) = 5.02$, $SE = 0.08$, $z = 19.2$, $p = 0.01$). n = 4 for Sham and n = 6 for Stim.
